# Fertility Preservation Techniques in Neuro-Oncology Patients: Protocol for a Systematic Review

**DOI:** 10.2196/44825

**Published:** 2023-05-08

**Authors:** Maia Osborne-Grinter, Offorbuike Chiamaka Bianca, Jasleen Sanghera, Chandrasekaran Kaliaperumal

**Affiliations:** 1 Centre for Surgical Research University of Bristol Bristol United Kingdom; 2 College of Medicine University of Ibadan Ibadan Nigeria; 3 College of Medicine University of Edinburgh Edinburgh United Kingdom; 4 Centre for Clinical Brain Sciences University of Edinburgh Edinburgh United Kingdom

**Keywords:** fertility, reproduction, central nervous system, CNS cancer, neuro-oncology, cancer, awareness, electronic database, tool, fertility preservation, patient, treatment, reproductive, oncology, infertility, in vitro fertilization, artificial insemination, human reproduction, reproduction, gynecology, gynecologist, assisted reproductive technology, gestational surrogacy, surrogate, fertility tourism, test tube baby, intracytoplasmic sperm injection

## Abstract

**Background:**

Advancements in cancer treatments have successfully improved central nervous system (CNS) cancer survivorship and overall quality of life. As a result, the awareness of the importance of fertility preservation techniques is increasing. Currently, a range of established techniques, such as oocyte cryopreservation and sperm cryopreservation, are available. However, oncologists may be hesitant to refer patients to a reproductive specialist.

**Objective:**

The primary aim of the proposed systematic review is to assess the best evidence for fertility preservation techniques used in patients with CNS cancers. It also aims to evaluate outcomes related to their success and complications.

**Methods:**

This protocol was produced in adherence with the PRISMA-P (Preferred Reporting Items for Systematic Review and Meta-Analysis Protocols). Electronic databases will be systematically searched to identify studies that meet our eligibility criteria. Studies will be included if they report at least one type of fertility preserving or sparing technique in male patients of any age and female patients aged <35 years. Animal studies, non-English studies, editorials, and guidelines will be excluded from the review. From the included studies, data will be extracted and synthesized by using a narrative approach and summarized in tables. The primary outcome will be the number of patients successfully undergoing a fertility preservation technique. The secondary outcomes will include the number of retrieved oocytes, the number of oocytes or embryos vitrified for cryopreservation, clinical pregnancy, and live birth. The quality of the included studies will be assessed by using the National Heart, Lung, and Blood Institute risk-of-bias tool for any type of study.

**Results:**

The systematic review is expected to be completed by the end of 2023, and results will be published in a peer-reviewed journal and on PROSPERO.

**Conclusions:**

The proposed systematic review will summarize the fertility preservation techniques available for patients with CNS cancers. Given the improvement in cancer survivorship, it is becoming increasingly important to educate patients about fertility preservation techniques. There are likely to be several limitations to this systematic review. Current literature is likely to be of low quality due to insufficient numbers, and there may be difficulty in accessing data sets. However, it is our hope that the results from the systematic review provide an evidence base to help inform the referral of patients with CNS cancers for fertility preservation treatments.

**Trial Registration:**

PROSPERO CRD42022352810; https://tinyurl.com/69xd9add

**International Registered Report Identifier (IRRID):**

PRR1-10.2196/44825

## Introduction

Central nervous system (CNS) cancers are the most prevalent cancers in 15- to 19-year-olds [1]. Infertility is one of the most common long-term issues experienced by young, female cancer survivors [2]. With advances in chemoradiotherapy and surgery meaning that survival from these cancers has considerably improved, there is an increased awareness of the impact that this has on cancer survivors’ quality of life [1]. CNS cancers can impact patients’ fertility in a variety of ways. Tumor infiltration, radiotherapy, and cranial surgery can all damage the hypothalamic-pituitary axis. In addition, chemoradiotherapy treatments can also be gonadotoxic, resulting in premature ovarian insufficiency and impaired fertility [3,4]. National Institute for Health and Care Excellence guidelines recognize the clinical importance of establishing fertility preservation techniques in those wishing to preserve their future reproductive potential [5].

There are several techniques available to women who are concerned about their future fertility, although many of these techniques have been considered experimental until recently. Embryo cryopreservation is the most established fertility preservation technique [6]. However, recent advances make oocyte cryopreservation an alternative option for those without a partner. Embryo or mature oocyte cryopreservation involves the hyperstimulation of the ovary to induce multiple follicles to grow [7]. Following retrieval, mature oocytes or embryos obtained via in vitro fertilization are cryopreserved and stored for use in the future [8]. For prepubertal girls, there is only one emerging technique available [9]. Ovarian tissue cryopreservation involves the transplantation of frozen-thawed ovarian tissue into the pelvic cavity after treating the CNS tumor. Although previous studies have shown successful results with this technique, there are concerns about tumor reseeding [10]. For patients undergoing radiotherapy, ovarian transposition, or oophoropexy, may be utilized as a fertility sparing method; the ovaries are surgically repositioned to be outside of the radiation field, thus minimizing their radiation damage [11]. For men, sperm cryopreservation is an established and effective technique for fertility preservation [12].

More young adults with CNS cancers see future family building as a possibility. However, oncologists may be hesitant to refer patients to a reproductive specialist, despite societal support and patient interest. This may be due to a reluctance to send mixed messages about prognoses, discomfort in discussing fertility or sexuality, and a lack of knowledge or time [12]. Our systematic review aims to summarize the available evidence concerning fertility preservation techniques used in the context of patients with CNS cancers.

## Methods

### Protocol and Registration

The protocol was developed in adherence with the recommendations of the PRISMA-P (Preferred Reporting Items for Systematic Review and Meta-Analysis Protocols) guidelines. The systematic review will also adhere to the recommendations of the PRISMA (Preferred Reporting Items for Systematic Reviews and Meta-Analyses) guidelines. The protocol has been prospectively registered on PROSPERO (registration number: CRD42022352810). Any changes in the protocol will be amended in PROSPERO and reported once the systematic review is complete.

### Eligibility Criteria (Study Designs and Types of Studies)

Studies will be included in this systematic review if they are a systematic review, case series, case report, interventional trial (nonrandomized and randomized controlled studies), cohort study, case-control study, or cross-sectional study reporting at least one type of fertility preservation technique among neuro-oncology patients. If multiple publications report results from the same patient cohort, then each study will be included, provided that distinct outcomes are reported. If these publications report duplicate outcomes, only the study with the largest population will be included. Grey literature will not be included in the review. Animal studies, non-English studies, editorials, and guidelines will be excluded from the review.

### Participants, Population, and Exposure

The review will focus on male and female patients of any age with a primary CNS tumor of any World Health Organization tumor grade. Such tumors may include medulloblastomas, ependymomas, astrocytomas, primitive neuroendocrine tumors, glioblastomas, germinomas, pineal tumors, gliomas, craniopharyngiomas, pineoblastomas, and CNS lymphomas. Participants must have received at least one type of fertility preservation or fertility sparing technique to be included. These may include medical therapy before chemotherapy, ovarian transposition, embryo cryopreservation, oocyte vitrification, ovarian tissue cryopreservation, or sperm cryopreservation. Participants who did not receive or were not referred for a fertility preservation technique will be excluded. If aggregate data prevent the identification of data meeting all the inclusion criteria, the paper will be excluded.

### Outcome of Interest

The primary outcome of the systematic review will be the number of patients successfully undergoing a fertility preservation technique. The systematic review is interested in fertility-related outcomes for patients undergoing fertility preservation techniques. These may be reported quantitatively or qualitatively. Therefore, the secondary outcomes include the success rates of different fertility preservation techniques, including, for example, the number of retrieved oocytes, the number of oocytes or embryos vitrified for cryopreservation, the rate of sperm cryopreservation, clinical pregnancy, and live birth. The number of oocytes retrieved reflects the effectiveness of fertility preservation protocols, and the number of oocytes or embryos vitrified for cryopreservation implies the potential for oocyte fertilization and embryo transfer [13]. A clinical pregnancy will be defined as at least one intrauterine pregnancy sac with yolk sac or original cardiac pulsations on a B-ultrasound examination. A live birth will be defined as the birth of a healthy infant not requiring admission to the neonatal intensive care unit, at 28 weeks gestation or more, during the follow-up period [14].

### Search Methods

We will perform a literature search of the following databases: PubMed, MEDLINE, Cochrane, Google Scholar, and Embase. A combination of Medical Subject Headings terms will be used to identify relevant articles. The search strategy will be developed in collaboration with a university librarian (Textbox 1). The search will be limited to articles published in the English language. To ensure the relevance of papers, the search strategy will be limited to papers published in the last 20 years (2002-2022). References will be hand-searched to identify other potential sources to be included. Grey literature will not be searched for the review.

**Provisional search terminology.**

**Search string**

- (“Fertility preservation“ OR “Ovary preservation“ OR “Oocyte preservation“ OR “Gonadal preservation“ OR “Sperm preservation“ OR “Semen preservation“ OR “Fertility cryopreservation“ OR “Ovary cryopreservation“ OR “Embryo cryopreservation“ OR “Gonadal cryopreservation“ OR “Sperm cryopreservation“ OR “Testicular cryopreservation“ OR “Ovariopexy“ OR “Ovarian transposition“ OR “Fertility sparing“ OR “Ovarian tissue cryopreservation“ OR “cryopreservation” OR “oocyte retrieval” OR “Ovary retrieval” OR “Gonadal retrieval” OR “OTC”) AND (“Brain tumour“ OR “Brain cancer“ OR “Brain neoplasm“ OR “Cerebral neoplasm” OR “Cerebellar neoplasm“ OR “Medulloblastoma“ OR “Ependymoma“ OR “Primitive neuroectodermal tumour“ OR “Primitive neuroectodermal cancer“ OR “Primitive neuroectodermal neoplasm“ OR “Astrocytoma“ OR “Glioblastoma“ OR “Germinoma” OR “Central nervous system tumour“ OR “CNS tumour” OR “Central nervous system cancer“ OR “CNS cancer” OR “Central nervous system neoplasm“ OR “CNS neoplasm” OR “Pineal tumour“ OR “Pineal cancer“ OR “Pineal neoplasm“ OR “Pinealoma” OR “Glioma“ OR “Craniopharyngioma“ OR “Pineoblastoma“ OR “CNS lymphoma”)

### Selection of Studies

The titles and abstracts of studies produced by the search will be collated by using reference management software, and duplicates will be removed. Two authors will jointly screen the titles and abstracts of the search results. The full texts of potentially relevant studies will then be retrieved and assessed against the predefined inclusion and exclusion criteria. When a consensus cannot be reached, a third reviewer will be consulted. When a full-text article cannot be obtained, the corresponding author of that article will be contacted. The selection process will be documented by using the PRISMA flowchart to report how studies were identified, screened, and included.

### Data Extraction, Management, and Synthesis

A standardized data extraction form will be developed and piloted before use. Data will be extracted from 10% of the selected studies by 2 reviewers independently. Concordance between the two reviewers will be statistically assessed by using the Kendall τ statistic. If concordance is met, the reviewers will each extract data from 50% of the remaining papers. If concordance is not met, discrepancies will be resolved via discussion, and data will be extracted from a further 10% of the studies and reassessed.

The following parameters will be extracted from each paper: primary author, publication date, study design, country, participant demographics (number, gender, age, duration of symptoms, type of brain tumor, tumor stage, age at treatment, radiation dose to ovaries [grays], radiation dose to the hypothalamic-pituitary axis [grays], and description of study participants), inclusion and exclusion criteria, the existence of pre-existing conditions affecting fertility, the type of neuro-oncology intervention(s), the type of fertility preservation, age at fertility preservation, the method of conception, the number of attempts at pregnancy, the outcome(s) measured and the value of the outcome(s) measured, and treatments (including teratogenic treatments) received during pregnancy. Individual study data will be combined and synthesized in a table. The following outcomes and end points may be included: pregnancy outcomes, the number of oocytes retrieved, ovarian tissue cryopreservation, follicle characteristics, sperm characteristics, rates of sperm cryopreservation, the number of metaphase-2 oocytes retrieved and vitrified, and neonatal outcomes. When data are missing, the authors will be consulted for further completing data.

Given the anticipated heterogeneity in study designs and outcomes, a meta-analysis may not be feasible. A narrative synthesis of the data will be performed. Tables will be used to summarize and present participant demographics, fertility preservation techniques, and outcomes. If possible, a subgroup analysis will be performed by stratifying women into age groups (<35 years and >35 years of age) and by pre-existing conditions affecting fertility (eg, polycystic ovary syndrome).

### Quality Assessment

All included studies will be assessed for quality and risk of bias by using the National Heart, Lung, and Blood Institute risk-of-bias tool for any type of study. The two reviewers will rate each domain of the included studies as having a low, high, or unclear risk of bias. These ratings will then be used to provide an overall quality score for the methodology of the article. Discrepancies between the two reviewers will be resolved through discussion with a third reviewer to achieve a consensus.

## Results

This is a protocol for a systematic review; therefore, the results cannot be presented. The results of the study will be submitted for publication in a peer-reviewed journal and presented on PROSPERO. The systematic review is expected to be completed by the end of 2023.

## Discussion

Given that cancer survivorship is increasing, it is becoming increasingly important to educate oncologists about fertility preservation techniques for patients who wish to preserve their fertility. Our systematic review aims to present a comprehensive summary of fertility preservation techniques used in neuro-oncology patients. To the best of our knowledge, this will be the first systematic review to evaluate these techniques in neuro-oncology patients. Current literature on the subject is largely comprised of small case series and is likely to be of limited quality due to insufficient numbers, difficulty in accessing data sets, and combined cohorts. This may limit the conclusions drawn in the systematic review. Our hope is that the review classifies the success rates of fertility preservation techniques and any complications experienced by the patients undertaking them.

Current guidance recommends that patients should be provided with information about the effects of cancer therapies on fertility before or during cancer treatment and that patients should be recommended for fertility preserving or sparing treatments as appropriate [15]. Through our review, we hope to provide clinicians with an overview of appropriate treatments to develop the understanding of treatments available. We also hope that the review facilitates the future development of frameworks to help guide clinician and patient discussions.

There are likely to be several limitations for the systematic review. The quality of current literature is likely to be low due to insufficient case numbers, difficulty in accessing data sets, and aggregate cohorts, limiting the conclusions that may be drawn from the systematic review. There is also likely to be significant heterogeneity in the outcomes reported in the studies. Therefore, it is likely that a meta-analysis will not be able to be performed. Moreover, there are multiple mediating variables, such as treatment type, tumor stage, and radiation dose, that must be considered when assessing fertility outcomes.

## Abbreviations

CNS: central nervous system
PRISMA: Preferred Reporting Items for Systematic Reviews and Meta-Analyses
PRISMA-P: Preferred Reporting Items for Systematic Review and Meta-Analysis Protocols

## Data Availability

Data are available on request.
